# Usher Syndrome

**DOI:** 10.3390/audiolres12010005

**Published:** 2022-01-11

**Authors:** Alessandro Castiglione, Claes Möller

**Affiliations:** 1 Audiology Department, Örebro University Hospital, 70210 Örebro, Sweden; claes.moller@regionorebrolan.se; 2 Audiological Research Centre, Faculty of Medicine and Health, Örebro University, 70182 Örebro, Sweden

**Keywords:** Usher syndrome, genetic hearing loss, ciliopathies

## Abstract

Usher syndrome (USH) is the most common genetic condition responsible for combined loss of hearing and vision. Balance disorders and bilateral vestibular areflexia are also observed in some cases. The syndrome was first described by Albrecht von Graefe in 1858, but later named by Charles Usher, who presented a large number of cases with hearing loss and retinopathy in 1914. USH has been grouped into three main clinical types: 1, 2, and 3, which are caused by mutations in different genes and are further divided into different subtypes. To date, nine causative genes have been identified and confirmed as responsible for the syndrome when mutated: *MYO7A*, *USH1C*, *CDH23*, *PCDH15*, and *USH1G (SANS)* for Usher type 1; *USH2A*, *ADGRV1*, and *WHRN* for Usher type 2; *CLRN1* for Usher type 3. USH is inherited in an autosomal recessive pattern. Digenic, bi-allelic, and polygenic forms have also been reported, in addition to dominant or nonsyndromic forms of genetic mutations. This narrative review reports the causative forms, diagnosis, prognosis, epidemiology, rehabilitation, research, and new treatments of USH.

## 1. Introduction

Human communication is, to a large extent, based on our “far senses”: hearing and vision [1]. The use of our “near senses”—smell, taste, and tactility—essentially becomes important when hearing and vision are impaired. Deaf-blindness is a combined vision and hearing impairment of such severity that it is difficult for the impaired senses to compensate for each other. Thus, deaf-blindness is a distinct disability that limits activities and restricts participation in society. It affects social life, communication, access to information, orientation, and the ability to move around freely and safely.

Usher syndrome (USH) is the most common deaf–blind syndrome, with 50% of deaf-blindness in persons younger than 65 years of age. It is a genetic condition that includes hearing loss, retinopathy (retinitis pigmentosa), and vestibular areflexia with different entities and onset [2,3,4]. It takes its name from the ophthalmologist Charles Usher, who described a large series of 69 affected patients from 40 families with hearing loss and retinopathy [5]. USH is inherited in an autosomal recessive pattern, and to date, nine causative genes have been identified and confirmed: *MYO7A*, *USH1C*, *CDH23*, *PCDH15*, and *SANS* for Usher type 1; *USH2A*, *ADGRV1*, and *WHRN* for Usher type 2; *CLRN1* for Usher type 3.

*CIB2* (USH1J) is no longer considered responsible for Usher syndrome and was recently excluded from Usher genes by Booth et al., 2018 (https://www.usher-syndrome.org/ accessed on 21 December 2021) [6].

Other genes and loci have been related to the disease; however, their roles are not clear and must be confirmed in further studies: *ESPN* (USH1M), *HARS* (USH3), *CEP78* (atypical Usher), *CEP250* (atypical Usher), *ABDH12* (USH3), and *ARSG* (atypical Usher), and three loci, namely, USH1E, USH1H, and USH1K. In addition, *PDZD7*, a modifier gene, is considered responsible for Usher type 2 when associated with mutations in other Usher genes, such as *USH2A*, *ADGRV1*, and *WHRN*. Nonsyndromic recessive and dominant hearing loss without retinopathy caused by USH genes has also been reported [7,8,9].

USH is the most common cause of syndromic hearing loss after Pendred syndrome [10,11,12] and is categorized into three clinical types with 14 subtypes, following causative mutations in different genes and loci. The subtype USH1A, previously identified and described in published papers, is no longer considered responsible for the syndrome and does not exist anymore; therefore, it was excluded from this review. Moreover, the subtype USH2B, which has not been confirmed, was also excluded [13,14,15,16]. As mentioned, the validity of *CIB2* is also being questioned by Booth et al., 2018 [6], even though it still appears in papers and conferences.

For the purpose of this review, it is important to consider that the syndrome is divided into three clinical subtypes (USH1, USH2, and USH3), identified by clinical investigation, and that different subtypes correspond to different responsible genes, identified by genetic investigation. However, genetic investigations are essential to confirm or reject the clinical diagnosis. The introduction of next-generation sequencing has improved the identification of new genes and mutations [17,18]. Nevertheless, confirming a clinical diagnosis requires comprehensive analyses, leading to a high number of variants whose roles should be defined and verified [19].

USH can be considered a ciliopathy [20,21] because it affects specific genes and proteins involved in various ciliary cell functions. Most genetic mutations cause the destruction and disruption of different structural proteins that play important roles in auditory, visual, and vestibular functions. However, whether USH should be considered a ciliopathy is still debated. The question concerns three main points: (1) terminology, (2) histology/cytology, and (3) pathophysiology.

**Figure 1:**
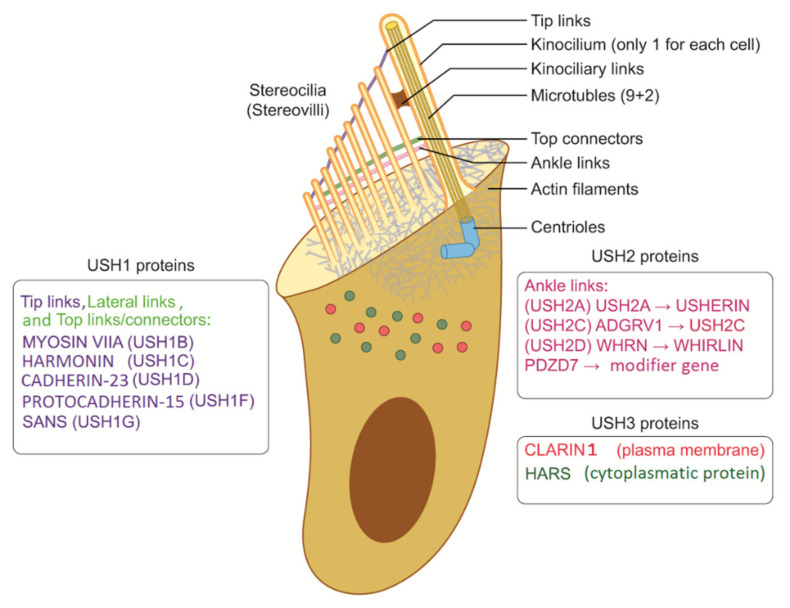
Schematic and simplified representation of a sensorineural cell in the inner ear.

**Figure 2:**
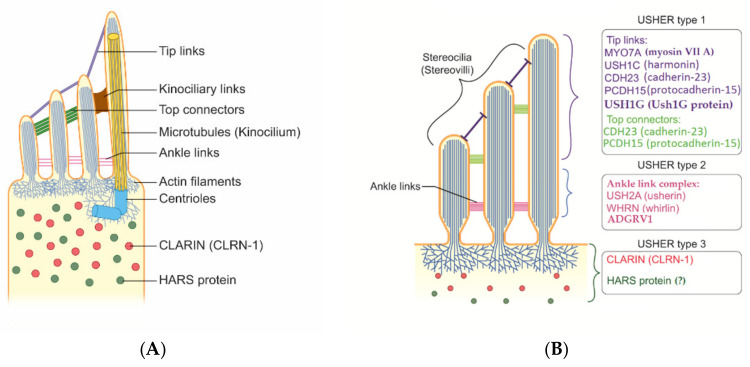
Simplified and schematic representation of structures, genes, and proteins involved in Usher syndrome. (**A**) The picture shows the main structures of a sensorineural inner ear cell. (**B**) Different genes and proteins have been identified, some responsible for different USH subtypes. It should be noted that clarin-1 protein (in red) is a four-transmembrane protein that is synthetized and collected in the cytosol and then moves to cellular membrane during maturation processes; in the membranes, clarin protein acts as a modulator of mechano-transduction. In the figure, clarin-1 is intentionally pictured inside the cytosol to keep it separated from other proteins. Hars protein (in dark green) is a cytoplasmatic enzyme. The role of *HARS1* gene in USH type 3 must be confirmed. HARS protein = histidine–tRNA ligase, cytoplasmic. Even if is not reported in the figure, myosin 7A seems to also interact with proteins of the ankle link complex.

The terminology might be quite confusing because the structures of inner ear cells have been called stereocilia (sing. stereocilium) and kinocilia (sing. kinocilium) [22]. Histology teaches us that stereocilia should be called stereovilli (sing. stereovillum) if made of actin as opposed to cilia (kinocilium) when made of tubulin (microtubules) (Figure 1). Nevertheless, several pathophysiological studies have demonstrated a number of interactions between actin filaments and microtubules, and the proper functions of actin filaments are essential for microtubules as well as stereovilli in cellular functions. From this perspective, when kinocilia are affected in the inner ear and photoreceptors are affected in the retina (inferring direct and indirect tubulin structures), USH can be considered a ciliopathy (Figure 1 and Figure 2) [23,24,25,26].

USH belongs to a group of ciliopathies affecting nonmotile or sensory cilia, which include Bardet–Biedl syndrome, Alström syndrome, Meckel syndrome, Joubert syndrome, and Leber congenital amaurosis (primary nonmotile ciliopathies). Despite sharing overlapping features, these syndromes are very different, and it is rare for the same gene to be involved in two different conditions. This suggests a strong specific causative relationship between the genotype and phenotype.

Even if both microtubules and actin filaments are involved in different ciliopathies, USH seems to interfere exclusively with sensory cells that express actin filaments in specific segments [27,28,29,30,31,32,33,34]. This may explain why the disease essentially affects the eyes and ears. Other conditions that widely affect microtubules in other organs, such as Alström syndrome, show a worse prognosis affecting several organs and tissues [35,36].

Despite symptoms following the distribution and expression of different proteins in different organs, the variability in expression of similar dysfunctions can be remarkable, even among siblings [37]. Penetrance is considered about 100% for all types, with the most severe forms (USH1) being characterized by congenital profound sensory hearing loss, bilateral vestibular areflexia, and early onset of retinitis pigmentosa (RP). USH2 is characterized by congenital moderate–severe but relatively stable sensory hearing loss, normal vestibular function, and RP, which is usually discovered in early teens. USH3 causes progressive sensory hearing loss, progressive vestibular loss, and RP in teens. Neuroradiological anomalies are usually not identified [38]; however, some studies reported minor abnormalities in the central nervous system. Interestingly, Schaefer et al., in 1998 [39], and Ciorba et al., in 2008 [40], reported a decrease in the volume of the cerebellum. The latter is involved in balance, posture, motor learning, language, and speech; thus, volumetric modifications may derive from decrease or alteration in neural inputs from eyes and ears [41]. However, further studies are required to confirm this association.

The scope of this narrative review is to provide an overview of the current clinical and genetic knowledge concerning the epidemiology, diagnosis, prognosis, rehabilitation, and treatment of USH. It intends to provide clinical features and genetical understanding, helpful to several specialists, to sustain a multidisciplinary approach and successful management of the syndrome. It is targeted to professionals, researchers, and students with interest in the field of otology, audiology, and genetics. Reported variants should be intended as examples and they do not refer to the prevalence of the variants. Unless otherwise specified in the text, they were selected from the literature for teaching purposes and to clarify what is eventually reported in the text. Figures and tables should be also intended as explicative, simplified, and helpful for readers in understanding the genetics, clinical aspects, and pathophysiology of USH. Genotype/phenotype correlations have been described following the most common accepted criteria available in the literature; however, exceptions are possible and partially reported in the text, even if they do not necessarily reflect the direct experience of the authors. Finally, the authors herein provide available solutions for the most effective rehabilitative approach in the management of USH following national guidelines, personal experience, and scientific literature; however, rehabilitation may be influenced by local laws, national guidelines, and health institutions with different approaches to the management of the syndrome. To the best of our knowledge, an international consensus statement on diagnosis, treatment, rehabilitation, and follow-up of USH has not been published to date.

## 2. Materials and Methods

A literature search was carried out in medical databases, such as “PubMed.” The keywords “Usher”, “syndromic hearing loss”, “blindness”, “retinitis pigmentosa”, “retinal dystrophy”, “ciliopathies”, and “deafness” were used, and 450 English articles from 1988 to 2021 were selected and analyzed. It is important to emphasize that this report is not a systematic literature review. The text has been formatted based on the following main categories: genetics, epidemiology, diagnosis, rehabilitation, treatment, and some future perspectives. Of the 450 articles, 128 full texts, best suited to our purposes, were selected and included in this review.

## 3. Genetics of USH

**Figure 3:**
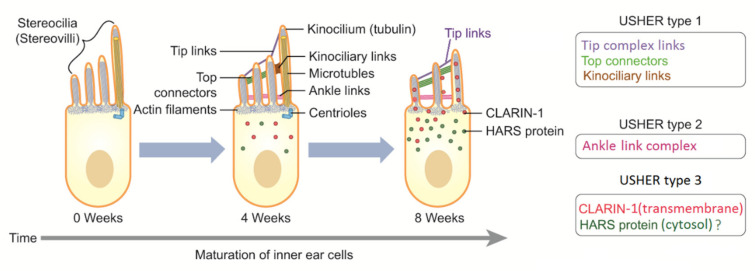
Simplified and schematic representation of the maturation process that involves sensorineural cells in the inner ear. Only one kinocilium and many different stereocilia (stereovilli) for each cell are presented. Kinocilia are intracellular, transmembrane, and extracellular proteins that drive maturation and orientation of stereocilia in the sensorineural epithelium. Inner and outer hair cells, and vestibular type II hair cells lose their kinocilium during maturation; the kinocilium disappears in the final form. Transient links degenerate, and tip links, top connectors, and ankle links assume their final functional form. This reorganization of tip links, transient lateral links, top connectors, kinociliary links, and ankle links refers to the normal functions of actin filaments and several Usher proteins. Centrioles are also very important in leading a vital process during cell life. Hearing loss and equilibrium disorders may be derived from mutations in different genes that express proteins involved in different levels and in different times of maturation of the sensorineural cells. Clarin-1 protein (in red) is a four-transmembrane protein that is synthetized and collected in the cytosol and then transported to cellular membrane during maturation processes. The role of *HARS1* gene in USH type 3 must be confirmed. HARS protein = histidine -- tRNA ligase, cytoplasmic. Myosin7A seems to interact with ankle link proteins in vitro. Its role in vivo is less clear. It has not been shown to be necessary in photoreceptors.

Even if kinocilium may present a motile structure (9 + 2), it is considered a nonmotile cilium, lacking inner dynein arms [42]. Degeneration of kinocilium under maturation of the ear leads to inner and outer hair cells without kinocilia. However, kinocilium is still present in the vestibular system (type I hair cell). Progressive structural reorganization of proteins involves lateral links, ankle links, tip links, and top connectors until the final mature structure is reached (Figure 3). Specialized structures are also present in photoreceptors (rods and cones), and they depend on the active connections between actin, Usher proteins, and tubulin structures. Genetic mutations causing hearing loss, vision impairment, and dizziness may involve all these processes.

In the eye, mutations in Usher genes are responsible for affecting light-sensing rods and cones, thus leading to the degeneration of the retina with pigment deposits, a condition named retinitis pigmentosa (RP). In RP, the retina’s light-sensing rods and cones slowly lose their properties and functions, starting at the outer edges.

Compromised photoreceptors, retinal pigment epithelium cells, and Müller cells are thought to be the cellular basis underlying the pathogenesis of RP [43,44,45].

Usher proteins are widely expressed in the retina and particularly in the connecting cilium, periciliary membrane, calyceal process, inner and outer segment, basal body, and synapse. Mutations are responsible for progressive degeneration and loss of functions of those structures. In particular, the rods are first involved in the process, thus leading to difficulties in seeing in darkness. Degeneration of cones often starts at the outer edges, causing a “tunnel view”, and subsequent degeneration causes loss of central vision [44].

To date, USH has been associated with 16 genes (Table 1): *ABHD12*, *ADGRV1*, *ARSG*, *CDH23*, *CEP250*, *CEP78*, *CIB2*, *CLRN1*, *DFNB31*, *ESPN*, *HARS*, *MYO7A*, *PCDH15*, *USH1C*, *USH1G*, and *USH2A*. In addition, one modifier gene (*PDZD7*) was identified in 2015. Three new loci, USH1E [27], USH1H [32,33], and USH1K [34], have been reported with mapping linkage; however, the gene responsible for these loci has not been identified yet (https://sph.uth.edu/retnet/sum-dis.htm#Agenes accessed on: 21 December 2021). Thus, genetic heterogeneity of this syndrome and some of the genes can also be found in nonsyndromic hearing and nonsyndromic RP forms. The large heterogeneity and rarity of USH often results in late or incorrect diagnosis in subjects with a mild phenotype [3]. Most USH-related genes express structural proteins with variable and exhaustible residual functions. This could explain the progression and variable onset of clinical findings. Even if USH is the most common genetic cause of hearing and vision loss, it should always be kept in mind that not all hearing and vision impairments are Usher syndromes.

To date, nine of the aforementioned genes have been identified and confirmed as causative genes: *MYO7A*, *USH1C*, *CDH23*, *PCDH15*, and *SANS* for Usher type 1; *USH2A*, *ADGRV1*, and *WHRN* for Usher type 2; *CLRN1* for Usher type 3.

Genetic heterogeneity can also arise from (1) various mutations in the same gene in different individuals, such as *MYO7A* alleles associated with differing phenotypes, (2) alleles responsible across different genes, or (3) phenotypic heterogeneity due to variable degrees of penetrance and expressivity. However, penetrance in USH type 1 and 2 is considered almost complete (99–100%).

Proteins involved in USH play different roles in hearing and vision. Most of them are structural proteins with mutual functions in hair cells and photoreceptors. Other proteins were found to play more of a functional role. In the ear, the structural proteins identified as components of the protein complex include Usher protein complex 1 (made of *USH1C*, *USH1G*/*SANS*, *PCDH15*, *CDH23*, and *MYO7A*) and Usher protein complex 2 (made of *USH2A*, *ADGRV1*, *WHRN*, and *PDZD7*). These complexes are involved in tip links, lateral links, top connectors, and ankle links (Figure 1 and Figure 2). In brief, mutations in proteins of complex 1 (tip links, lateral links, and top connectors) are generally responsible for USH1, mutations in ankle links (complex 2) are responsible for USH2, and mutations in *CLRN1* and *HARS* are responsible for USH3 [45,46]. However, the roles of other genes should be confirmed. The protein myosin-VIIa interacts with ankle link proteins, and its role in supporting USH2 proteins is widely reported in vitro. However, its role in vivo is less clear. Zou et al., in 2017, reported that only myosin VIIa is indispensable for USH2 complex assembly at ankle links, indicating the potential transport and/or anchoring role of myosin VIIa for USH2 proteins in hair cells. However, myosin VIIa is not required for USH2 complex assembly in photoreceptors [47].

Protein complexes have also been identified in the eye: when mutated, USH1 proteins seem to affect calyceal processes and USH2 proteins are involved in periciliary complex. Clarin-1 is a four-domain transmembrane protein that regulates ion channels in the cellular membrane of sensorial cells in the retina and inner ear. Therefore, mutations in *CLRN1* gene affect ion channels in synapses and stereocilia.

Mutations in the Usher genes inhibit the expression of proteins that are important for maintaining the integrity of protein structures, thereby resulting in the absence of Usher complex.

Here follows a short review of all suspected genes potentially involved in Usher syndrome; information on the function and expression for each gene is also provided. When possible, phenotype–genotype correlations for different genes are described.

### 3.1. USH1

#### 3.1.1. *MYO7A*—Myosin VIIa, USH1B

This gene is one of the most common and is frequently involved in Usher syndrome. It belongs to a superfamily of motor genes that encodes large proteins, the so-called myosins. They are known to play a role in many motile functions in mammals. Different myosins have different functions in many organs and they may interact with actin filaments to provide specific and specialized functions in hearing and vision. Some myosins (e.g., *MYO6*, *MYO7A*, or *MYO15*) seem to be involved in hearing loss when mutated, while only *MYO7A* has been associated with USH. Structural proteins play important roles in the functions and movements of actin cilia. In the inner ear motor, some Usher proteins are hypothesized to be part of an Usher protein network constituted by *USH1C*, *USH1G*, *CDH23*, and *MYO7A*, which mediate mechano-transduction in cochlear hair cells, vestibular hair cells, and photoreceptors (primarily the rods). Furthermore, mutated *MYO7A* causes USH1B, the most common form of USH1. Mutations in this gene can also be found in rare cases of nonsyndromic hearing loss (*DFNA11* and *DFNB2*). Myosins are mechano-chemical proteins characterized by the presence of domains that interact with other proteins, with structural properties that allow ciliary movements in actin filaments.

Lastly, *MYO7A* encodes an unconventional myosin [48,49,50,51] that moves along actin filaments, interacting with specific macromolecules transporting different cargo, for example, opsin along the connecting cilium to the outer segment in the rod cells. The interactions with actin filaments are necessary for normal function. *MYO7A* is located on chromosome 11q and is widely expressed in the hair cells of the inner ear, human kidney, liver, and retina, but not in the brain or lymphocytes. Weil et al., in 1996, determined that the *MYO7A* gene contained 48 coding exons and 2215 amino acids [52]. Interestingly, mutations in *MYO7A* gene can be responsible for nonsyndromic recessive or dominant hearing loss, as well as for syndromic forms, such as in Usher syndrome. Different sites of mutations in the genetic sequence suggest some residual functions for mutated proteins or degeneration due to the precipitation of parts of protein (dimerization).

Reported pathological variants are p.R150X (USH 1B), p.Q234X (USH 1B), p.R212H (USH1B), p.R212C (USH1B), C168X (USH1B), p.G214R (USH1B), p.A397D (USH1B), p.R244P (DFNB2), p.M599I (DFNB2), p.R395H (DFNB2), C31X (USH1B), C628X (USH1B), and 9-BP DEL exon 22 (DFNA11) [3,53,54,55]. Interestingly, it should be noted that truncating mutations are mostly responsible for USH.

As widely reported in the literature, *MYO7A* mutations among Usher patients are responsible for congenital severe-to-profound hearing loss, equilibrium disorders, and early onset of RP. Exceptions are reported where the most of pathological variants are usually responsible for profound congenital hearing loss with the absence of vestibular function. RP is also severe and rapidly progressive, with early onset [56,57]. Bilateral cochlear implantations (CIs) have provided strong evidence of successful rehabilitation among USH, DFNB2, or DFNA11. In some cases, it is possible that those three diagnoses are expressions of the same condition with different expressivity or mild phenotypes that do not allow a correct diagnosis. For example, it has been reported that patients with DFNB2 have been subsequently investigated and diagnosed for mild RP. Nevertheless, “age at surgery” is the most important prognostic factor [58,59,60]. Ineligible cases till surgery or poor outcomes of CIs may benefit from tactile and sign language [61].

#### 3.1.2. *PCDH15*—Protocadherin-15, USH1F

This gene encodes a structural protein called protocadherin and is a member of the cadherin superfamily. Protocadherin belongs to the family of integral membrane proteins that mediate calcium-dependent cell–cell adhesion. It plays an essential role in the maintenance of normal retinal and cochlear functions. It is a crucial part of the tip link structure (tip link complex) and interacts with cadherin-23 to form tip links that are extracellular filaments that connect stereovilli in the inner ear. The tip link complex is located in the apical part of the hair cells. Mutated proteins cause defective tip links and affect the functions of the stereocilia, particularly the mechanoelectrical transduction [62]. Furthermore, mutations in this gene are responsible for DFNB23 and Usher type 1 subtype F [63]. It is thought that hypomorphic alleles and less severe mutations result in DFNB23, whereas severe mutations may result in USH type 1F. Protocadherin can also be a vital part of other connectors between the stereovilli and kinocilium, even during cell maturation, such as the horizontal top connectors (apical lateral links) and transient lateral links. These last links are usually not so evident in matured hair cells, but they may influence hearing function under the maturation process and lead to dysfunctional hair cells, resulting in severe-to-profound congenital hearing loss. Exceptions are possible even though rarely reported. In 2021, Wafa et al. reported one case of USH1F affected by moderate hearing loss, bilateral vestibular hypofunctions, and despite RP, relatively preserved visual acuity and visual field [57].

*PCDH15* encodes for 33 exons and 1995 amino acids. R245X is considered a founder mutation responsible for approximatively 50–60% of USH1 in the Ashkenazi Jewish population [64]. Other reported mutations are p.R3X (USH1F) [65], c.del1471T (USH1F) [63], p.K808X (USH1F) [7], p.R134Q [66], and S647X (USH1F).

Chen ci Wu, in 2015, reported that children with *PCDH15* mutations were associated with poor CI performances and should be screened through next-generation sequencing for prognostic purposes to explore cochlear implantation outcomes [67].

*PCDH15* is widely expressed in the brain and nerves, and it is thought that mutations in *PCDH15* also involve the neural partition of the cochlea. In 2021, Nisenbaum et al. included *PCDH15* mutations among children that had no successful outcome post CI. This would be a strong recommendation for genomic screening among CI candidates with USH before surgery [68]. These patients may benefit from hearing aids; however, they should learn tactile signing and sign language in their early stages of life. Nevertheless, further studies are required to confirm poor CI outcomes among patients with *PCDH15* mutations.

#### 3.1.3. *USH1C*—Harmonin

This gene encodes harmonin, an anchoring scaffold protein that is also hypothesized to be a part of the Usher protein network. The gene was identified in the short arm of chromosome 11 [51,69,70]. Mutations in this gene are responsible for DFNB18 and Usher type 1C, which is the most common cause of USH in French Canadian and Acadian families in Louisiana, USA [70,71].

#### 3.1.4. *USH1G (SANS)*—USH Type-1G Protein, USH1G

Localized on the long arm of chromosome 17, this gene is responsible for the transcription and encoding of a small protein named USH type-1G protein. The encoded protein is an anchoring/scaffolding protein that interacts with harmonin and is also a part of the Usher protein network in hair cells. The gene is found in the USH1G subtype [72,73,74].

#### 3.1.5. *CDH23*—Cadherin-23, USH1D

This gene is a member of the cadherin superfamily, whose genes encode calcium-dependent cell–cell adhesion glycoproteins. These are transmembrane proteins that are thought to be involved in stereocilia organization and hair bundle formation. It is involved in tip links, top connectors, and lateral links, and when mutated, is responsible for USH1. The gene *CDH23* is located in 10q21–q22, a region containing the human deafness loci DFNB12 and USH1D. *CDH23* contains 69 exons and encodes for 3354 amino acids. *CDH23* is required for proper organization development of stereocilia in the cochlea and vestibule. It is also part of the Usher protein network, USH1D, which is the second most common genetic type of USH1.

Pathological variants responsible for USH1D are p.Q1496H, p.R1746Q, p.E1006K, p.E2520K, c.2289 + 1G > A, and p.Q492X [7,57].

The following variants are responsible for DFNB12: p.D1243N, p.D1400N, p.D2148N, p.D1341N, p.V586M, p.R301Q, and p.P240L. Cochlear implantation has provided successful outcomes worldwide.

#### 3.1.6. *CIB2*, USH1J?

This gene is located on chromosome 15q25.1 and encodes a calcium-binding regulatory protein. These proteins are involved in intracellular calcium homeostasis. It is also involved in the maintenance of photoreceptor and hair cells. Mutations in this gene are responsible for DFNB48 and USH1J. It was previously reported to be responsible for USH1H but was subsequently identified as a different gene and therefore named USH1J, with the gene in the locus USH1H still unknown. The protein localizes to the stereocilia of inner ear hair cells and photoreceptors and is postulated to be a component of the Usher protein network [32,75]. Its role has recently been revaluated and it is no longer considered an usher gene even if it is still reported to be responsible for USH1J [6].

#### 3.1.7. *ESPN*—Espin

This gene encodes a multifunctional actin-bundling protein called espin. It plays a major role in regulating the organization, dimensions, dynamics, and signaling capacities of actin filament-rich microvilli in mechanosensory and chemosensory cells. It is required for the assembly and stabilization of stereocilia in parallel actin bundles. Mutations in this gene are associated with autosomal recessive sensorineural deafness (DFNB36) with or without vestibular involvement and USH1M [50,76]. However, its role in USH must be confirmed with further studies.

#### 3.1.8. USH1E, USH1H, and USH1K

Three new loci were identified in different families, after mutations in known genes for USH were excluded. They were named USH1 E, H, and K, and cryptogenic studies have revealed that these three loci map to chromosomes 21, 15, and 10, respectively (Table 1). To date, no responsible genes have been identified [27,32,33,34]. Further studies are required to confirm the role of these loci in Usher syndrome and to identify the responsible genes.

### 3.2. USH2

#### 3.2.1. *USH2A*—Usherin

Usherin is a protein encoded by *USH2A*, which is localized to the long arm of chromosome 1. It is a structural protein expressed in the basement membranes of the cochlea and retina. Usherin potentially plays an important role in the homeostasis of eye and ear functions. It is also thought to be a part of other genes in the Usher protein network. Furthermore, Usherin is necessary for the function and organization of the periciliary membrane complex in photoreceptors by regulating intracellular protein transport. The USH2 protein network consists of *USH2A*, *GPR98* (*ADGRV1*), *WHRN*, and *PDZD7* [77]. The proteins are an essential part of the ankle links, extracellular connections between stereovilli localized at their base (Figure 2 and Figure 3). Mutations in *USH2A* are usually responsible for moderate-to-severe hearing loss and because of the absence of progression, bilateral hearing aids are usually beneficial. *USH2A* mutations are the most frequent worldwide. Reported variants are p.Cys934Trp, p.(Thr65Ilefs*80), p.R34X, p.R63X, p.W3955X, and p.R4608X [2,7,78,79,80,81]. Hearing loss is generally identified in childhood; however, RP occurs often during the second decade of life, leading to delay in diagnosis of USH. Truncating mutations seems to be associated with earlier onset and the worst prognosis [80]. Cases affected by mild-to-profound hearing loss have been reported [7,57].

#### 3.2.2. *ADGRV1* (*GPR98* or *VLGR1*)—Adhesion G-Protein Coupled Receptor V1, USH2C

The gene *ADGRV1*, also known as *GPR98* or *VLGR1*, encodes a calcium-binding G protein-coupled receptor. The encoded protein contains a 7-transmembrane receptor domain, binds calcium, and is widely expressed in the central nervous system, eye, and ear. It has an essential role in the development of hearing and vision. The protein is required for the formation of the ankle link complex in the hair cells (Figure 2). In addition, it is involved in different processes, such as photoreceptor cell maintenance and sensory perception of light stimuli. The protein is localized in the plasma membrane, at the ankle region of the stereocilia and in photoreceptors, at periciliary membrane complex, the apical inner segment that surrounds the connecting cilia. Mutations in this gene have been associated with USH2C and familial febrile seizures [82,83,84].

#### 3.2.3. *WHRN*—Whirlin, USH2D

This gene is thought to function in the organization and stabilization of stereocilia (stereovilli) elongation and actin cystoskeletal assembly. Mutations in this gene have been associated with autosomal recessive nonsyndromic deafness and USH. Whirlin (*WHRN*) is a protein-coding gene. Diseases associated with *WHRN* include deafness, autosomal recessive hearing loss (DFNB31), and USH2D [44,45,85,86]. It is necessary for elongation and maintenance of inner and outer hair cell stereocilia and is involved in the maintenance of the hair bundle ankle region (ankle links), which connects the stereocilia of the hair cells. In the retina, *WHRN* is important for the maintenance of a periciliary membrane complex that seems to play a role in regulating intracellular protein transport in photoreceptors [87,88,89]. Lin et al. recently reported that whirlin interacts with the EPS8 protein in the tip complex [85]. This interaction is mediated by the MYO15 protein, which is responsible for DFNB3 when mutated. *EPS8* encodes an epidermal growth factor protein that is also reported to be responsible for DFNB102 and syndromic hearing loss (branchio-oto-renal syndrome) [44,45,85,86]. Thus, the roles of genes encoding different USH protein networks are still unclear.

#### 3.2.4. *PDZD7*, A Modifier Gene?

This gene encodes a ciliary protein homologous to proteins that are mutated in patients with USH, and mutations and translocations involving this gene have been proposed to be associated with USH2A and DFNB57 [90]. It is considered a modifier gene and contributor to digenic USH [88]; however, its role still needs to be fully understood. In 2015 and 2016, respectively, Booth et al. [91] and Vona et al. [92] reported biallelic variants, without mutations in USH genes associated with nonsyndromic hearing loss, thus confirming their role in DFNB57.

### 3.3. USH3

#### 3.3.1. *CLRN1*—Clarin-1, USH3A

This gene encodes a protein (clarin) that may be important in the development and homeostasis of the inner ear and retina. It is thought to play a role in the excitatory ribbon synapse junctions between hair cells and cochlear ganglion cells and presumably also in analogous synapses within the retina. It acts as a modulator of transduction activity. The gene was found to be connected to the USH3A subtype [93,94,95,96,97,98]. A founder mutation, p.Y176X, is present in Finland [99], but linkages to the USH3 region have also been found in families from the USA and Sweden. Founder mutation among Ashkenazi has been reported as being c.144T > C; p.N48K [97]. *CLRN1* mutations have also been reported in a potential digenic deafness associated with *MYO7A* [100,101,102]. Clarin-1 is a transmembrane protein localized in the cellular membrane; however, some studies have localized this protein in other cytoplasmatic structures, such as Golgi and endoplasmic reticulum. USH3A has been associated to good cochlear implantation outcomes [103].

#### 3.3.2. *HARS1*—Histidine—tRNA Ligase, Cytoplasmic, USH3B?

*HARS* is located on chromosome 5q31 and encodes an aminoacyl-tRNA synthetase. Mutations in this gene have been hypothesized to be linked to USH3B and Charcot–Marie–Tooth Disease (axonal, Type 2 W). The protein encoded by this gene is a cytoplasmic enzyme that belongs to the class II family of aminoacyl-tRNA synthetases. The enzyme is localized in the cytoplasm, and it is responsible for the synthesis of histidyl-transfer RNA, which is essential for the incorporation of histidine into proteins [46].

Few data are available in the literature on cochlear implantation outcomes among patients with *HARS* mutations. In 2012, Puffenberger et al. reported partial restoration with hearing aids or cochlear implant [46]. Its role in USH3 should be confirmed in further studies.

#### 3.3.3. *ABHD12*—Lysophosphatidylserine Lipase ABHD12, USH3?

This gene is located on the short arm of chromosome 20 and is an acronym for polyneuropathy, hearing loss, ataxia, retinitis pigmentosa, and cataract due to an inborn error in endocannabinoid metabolism. The *ABHD12* gene encodes an enzyme that is involved in the hydrolysis of 2-arachidonoyl glycerol (2-AG), the main endocannabinoid lipid transmitter that acts on cannabinoid receptors CB1 and CB2. It was originally localized in Norwegian families, and in 2012, a homozygous *ABHD12* mutation was identified and reported in a Lebanese family with USH3-like findings and cataracts [104]. Its role in Usher syndrome is not clear, and further studies are necessary to confirm these statements.

### 3.4. Atypical Usher or Unclear Role

#### 3.4.1. *CEP250*—Centrosome-Associated Protein CEP250, Atypical Usher

New forms of a potential “atypical USH,” with mild sensorineural hearing loss and different retinal degeneration, have recently been found in some families. Centrosomal proteins are proteins that are localized in the centrosome or centriolar satellites. Centrosomes and centriolar satellites seem to play important roles in the cellular cycle and are responsible for the correct matching of chromosomes during mitosis and meiosis. However, they are also involved in the maintenance of regular function and organization of primary or motile cilia, which are located in the basal body of cilia and microtubules. These proteins may be considered structural proteins and their role can explain their involvement in several ciliopathies [105,106], affecting several organs that express motile and primary cilia. The *CEP250* gene (chr. 20q) encodes a core centrosomal protein required for centriole–centriole cohesion, thus playing an important role during the interphase of the cell cycle.

Diseases associated with *CEP250* mutations include cone–rod dystrophy, hearing loss 2, and USH. Its possible role in USH is not yet fully understood [107].

#### 3.4.2. *CEP78*—Centrosomal Protein of 78 kDa, Atypical Usher

This gene encodes a centrosomal protein of 78 kDa that is required for the regulation of centrosome-related events during the cell cycle and is required for ciliogenesis. Naturally occurring mutations in this gene cause defects in primary cilia that result in retinal degeneration and sensorineural hearing loss. This can be associated with photoreceptor degeneration hearing loss and may be a new form of USH [108,109,110].

#### 3.4.3. *ARSG*, Arylsulfatase G, Atypical Usher

*ARSG* encodes the protein arylsulfatase G, which hydrolyzes sulfate esters from sulfated steroids, carbohydrates, proteoglycans, and glycolipids. They are involved in hormone biosynthesis, modulation of cell signaling, and degradation of macromolecules. This protein is localized in the lysosomes. In 2018, Khateb et al. reported mutations in this gene in a family with “atypical” Usher [111]. Subsequently, it has been described as Usher 4, but this new entity has not been widely reported and accepted to date [111,112,113,114]. The introduction of a new clinical type USH4 is not widely accepted, and further studies are required to confirm these hypotheses.

## 4. Epidemiology

USH is a rare condition in the general population. However, it should be considered a common condition for professionals, geneticists, ophthalmologists, otolaryngologists, and audiologists, since mutations in genes responsible for Usher are the most common cause of ciliopathies and deaf-blindness. The prevalence rate is not well described globally, and large variations in prevalence have been reported, probably due to a lack of diagnostic and rehabilitation resources. Nevertheless, the prevalence has been estimated with greater accuracy in Great Britain, Sweden, Norway, Finland, Denmark, Italy, the Netherlands, Australia, and the United States. In Asia and Africa, only a few reports have been published, but the prevalence in most countries and ethnic groups has been estimated. Considering these discrepancies, the global estimation is 1/30,000 inhabitants, which should be considered as a low average because several studies have reported a wide range of prevalence that varies from 1 to 17 per 100,000 inhabitants [115]. A Swedish USH database with clinically and genetically confirmed USH, as managed by the authors (C. Möller personal communication), reports a prevalence of 10/100,000 inhabitants. USH accounts for ~9.2% of congenital profound deafness in children [116]. Therefore, USH is the most common cause of syndromic hearing loss after Pendred syndrome. The prevalence of different clinical types and genetic mutations shows great variability. It is widely accepted and documented that USH2 is the most common form (60%), with the *USH2A* gene accounting for 80–90% of cases [78]. In USH1, there is greater heterogeneity and variation between different countries and ethnic groups. The reason for this is the prevalence of previous geographical isolates such as in northern Scandinavia (*USH1B* and *USH1D*) and Louisiana, USA (*USH1C*), where USH1 is more common than USH2. Furthermore, isolated communities and cultural causes often result in deaf-by-deaf marriages [78]. USH3 is rare globally, except in Finland, where the prevalence is ~40% for USH3, 34% for USH1, and only 12% for USH2 [99]. Sweden reported higher prevalence rates of mutations responsible for USH1 than those of mutations responsible for USH2 [117]. This is, however, slowly changing, with a more even distribution between USH1 and 2. An example of an ethnic isolate is the Usher found in Ashkenazi Jewish affected families, where USH3 accounts for 40% of all USH cases [118]. An overview of the epidemiology is also displayed in Table 2 [2,3].

## 5. Diagnosis and Prognosis

Clinical USH diagnosis is essentially based on the association between hearing loss and RP, where hearing loss is congenital, and RP is apparent in childhood or young adulthood (Table 3). To date, no USH type has been discovered with late-onset hearing or vision loss. A combination of genetic and clinical diagnoses is required. The next-generation sequencing panel list for genetic hearing loss worldwide generally includes all known candidate genes for all types of USH. In most industrialized countries (2021), a clinical diagnosis is followed by a genetic confirmation, but this will probably change where next-generation sequencing with broad gene panels will be used after a neonatal discovery of hearing impairment.

### 5.1. Hearing Loss

**Figure 4:**
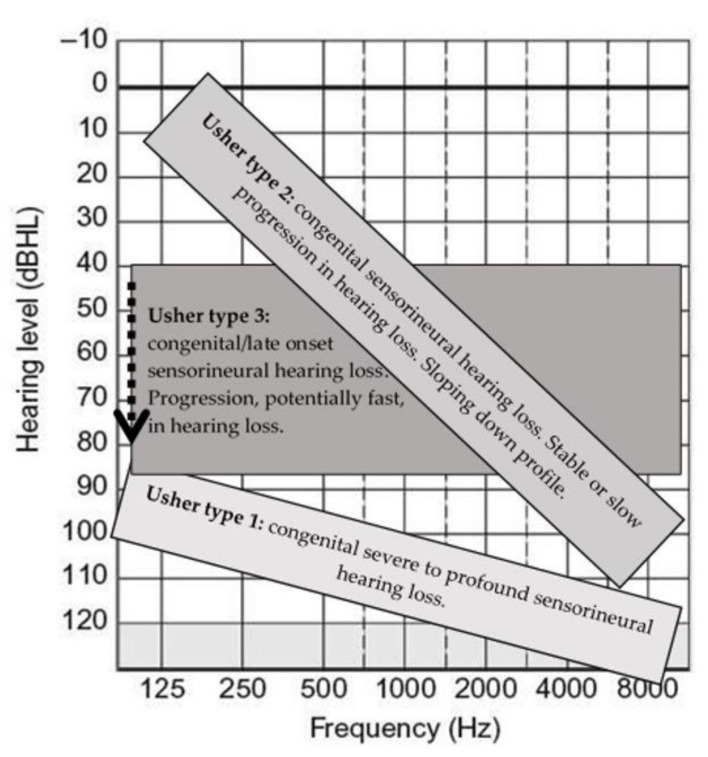
Usher Syndrome: audiometry profiles of patients with Usher type 1, 2, and 3. Hearing loss is bilateral, symmetric, and sensorineural for all types.

A neonatal audiological diagnosis should include otoacoustic emission (OAE), auditory brainstem response (ABR) with threshold measurements, auditory steady-state response (ASSR), and stapedial reflexes to determine the degree of hearing loss and rule out auditory neuropathy. The localization of hearing loss in USH is cochlear, with loss of both outer and inner hair cells. Later in childhood and during the life course, the addition of pure tone audiometry and speech audiometry should be included. The differences between USH1, USH2, and USH3 are as follows (Figure 4):

USH1: Congenital profound bilateral deafness.

USH2: Congenital moderate-to-severe bilateral hearing loss. In most cases, stable hearing loss occurs during the first 3–4 decades. Hearing loss has a sloping pattern: in most cases mild to moderate in the low frequencies and severe to profound at higher frequencies.

USH3: Congenital hearing loss, which is usually mild during childhood with a typical sloping pattern. The hearing loss is more variable, which differs from USH2 in that it is progressive with acquired severe-to-profound hearing loss usually in the third decade.

### 5.2. Balance

USH1: Vestibular investigation is an essential part of the diagnosis since all children suffer from bilateral vestibular areflexia. In small children, a rotatory test in an office chair using video Frenzel goggles reveals absent horizontal nystagmus during rotation. Assessment of motor milestone development shows delayed gross motor development with hypotonia as neonates; the children show delays in sitting and crawling and usually have a walking age of >18 months. Later, during childhood, they present difficulties in walking balance beams and difficulties in learning to ride a bicycle.

USH2: Vestibular function is normal with normal motor milestones.

USH3: Balance function is usually normal during childhood and early adulthood and is decreased later.

### 5.3. Vision Loss

Vision loss is caused by a degenerative retinal disorder called “retinitis pigmentosa.” This name goes back to the time at which the findings in the retina were thought to be related to inflammation. Today, we know that RP in USH is a slow retinal degeneration that initially affects the rods. RP in USH should not be compared with other forms of RP since the progression in USH is much slower than in other RPs. There are essentially two known types of photoreceptor cells (rods and cones), and ganglion cells involved in RP, thus resulting in progressive vision impairment. Rods are primarily responsible for nighttime vision, so-called scotopic conditions. The contribution of ganglion cells in RP remains unclear. The rods are located in the periphery of the retina and are also responsible for peripheral vision, while cones contribute to daytime and central vision (photopic conditions). Cones are also responsible for the color vision. RP is quite similar in all Usher types, and individual variation is usually greater than the variation between types. Vision loss in childhood is mild and may go undetected during the first 10 years. The slow degeneration of rods results in minor problems with contrast vision and light sensitivity. Progressive problems with night vision can be detected at around 8–10 years of age. Visual diagnosis should be made as early as possible. It is possible to detect dysfunction of the rods by electroretinography at an early age. Fundoscopy and testing of the visual field in small children are usually not sufficient to detect RP in USH [119].

The next symptom to appear is the so-called scotomas, which are small blind spots where small objects might be difficult to see. This is usually not noted by younger children who obviously do not comprehend how their vision is different from others. However, later in teenage and young adulthood, the degeneration of the rods increases, resulting in large peripheral visual loss and “tunnel vision.” At this stage (20–30 years), the night vision capability is extremely poor. In most cases, cataracts develop around 30–40 years. Several people with USH have preserved some central vision throughout most of their lives, although with visual fields of only 5–10 degrees [120,121].

In making a correct clinical and genetic diagnosis of USH1, it is important to keep in mind that there exist rare cases characterized by a nonsyndromic deafness with “typical” USH1 mutations.

### 5.4. Prognosis

USH1: To date, children with USH1 have often been misdiagnosed as having a nonsyndromic profound deafness. Today, in most industrialized countries, deafness is discovered through universal neonatal screening. Since deafness in USH is always bilateral, early cochlear implantation should be performed (6–12 months) to provide hearing and spoken speech [122]. It is important to have different modes of communication, and it is the authors’ belief that sign language is also of great importance. Since most adults with USH1 use sign language, it is noteworthy that visual sign language in later USH1 sometimes has to be changed into tactile sign language. The balance function is hampered, but early motor training can be compensated for by balance training, enforcing the use of proprioception and vision. The vision loss is progressive, as discussed earlier. This will gradually affect communication modalities because visual sign language with more than one person is extremely difficult with a visual field of 5–10 degrees. Deaf-blindness makes it difficult for the impaired senses to compensate for each other, which limits activities and restricts participation in society. It will also have an impact on social life, communication, access to information, orientation, and the ability to move around freely and safely.

USH2: The hearing loss in USH2 is so severe that it can be detected by neonatal hearing screening. The hearing loss should be treated as soon as possible after diagnosis with bilateral hearing aids (2–3 months). It is quite easy to fit children with USH2 since hearing loss during childhood and young adulthood is relatively stable. Most subjects with USH2 harbor mutations involving the Usherin gene (*USH2A*). It has recently been evident that the deterioration of hearing to some extent is dependent on different mutation patterns. Some older adults have shown a combination of congenital hearing loss and age-related hearing loss, which has resulted in profound deafness and cochlear implantation [123]. The degree of hearing loss has also been shown to differ among siblings with the same mutation [124].

RP in USH2 shows slow progress in USH1. In some studies, there appears to be a minor difference when the visual field restriction is slightly more rapid in USH1 than in USH2. The progression of vision loss is difficult to encompass because the ability to lip read is becoming increasingly difficult, especially in bad light conditions [121].

USH3: The clinical course in USH3 is more variable than that in USH1 and 2. Hearing loss in childhood is variable but shows a rapid progressive hearing deterioration at 20–30 years. The vestibular function is also decreasing and, in combination with vision loss and imbalance (especially at night), might result in falls and fractures. Unlike the rapid progression of hearing loss, RP does not seem to differ from USH1 and 2 [102].

## 6. Usher Type 4?

Recently published articles have suggested a new type of USH. To the best of our knowledge, the existence of a fourth type of Usher is not clear and must be confirmed. Usher type 4 has been proposed in a new study among five subjects from three families with atypical forms of Usher. However, further evidence is required to support the introduction of this new type as an atypical Usher form [125].

## 7. Rehabilitation

Deaf-blindness, as well as USH, is the result of the progression in various combined vision and hearing impairments to such an extent that it is difficult for the impaired senses to compensate for each other. However, deaf-blindness is a distinct entity and should not be rehabilitated as a simple coexistence and summation of two single impaired senses (hearing and vision). Since USH limits activities and restricts full participation in society, it also affects social life, communication, access to information, orientation, and the ability to move around freely and safely. To compensate for this loss, the tactile sense becomes important. Furthermore, because vision loss is progressive, the mode of communication must be continuously changed. Communication will always be time-, cognitive-, and energy-consuming. Impaired vision and hearing function increases the need to make use of other sensory stimuli (i.e., tactile, kinesthetic, haptic, smell, and taste). It limits access to distance information and creates a need to rely on information within the near surroundings. Extensive research in gene therapy is at hand; however, to date, there is no effective treatment beyond clinical trials in preventing or treating hearing, vision, or vestibular loss. Correct and early rehabilitation can prevent many other physical and psychological co-morbidities following progressive deaf-blindness.

### 7.1. Hearing

Most children with USH1 in developed countries are offered cochlear implants (CIs) with high efficacy. In Sweden, the majority of subjects with USH3 are offered CI, usually around 30–40 years [126]. Some persons with USH1 who make use of sign language but not spoken language have received CI, not to obtain speech but due to severe visual loss or blindness, which could enable them to achieve sound localization (i.e., helpful in traffic).

The hearing loss in USH2 is stable in most cases, and the linguistic outcome is good when hearing aid fitting and audiological rehabilitation start early. The difference in audiological rehabilitation between people with deaf-blindness and people with nonsyndromic hearing loss is that humans always use a combination of hearing and vision, as symbolically illustrated by “One hears with the eye and sees with the ear.” The authors believe that children with USH should obtain sign language as a second language, to have an alternative mode of communication if blindness should occur and tactile sign language has to be used in compliment with hearing.

### 7.2. Vision

Early diagnosis using electrophysiology will ensure correct and early rehabilitation. Once the diagnosis is obtained, yearly visual examinations should be performed, and pediatric ophthalmologists and optometrists should pay attention to contrast sensitivity, light sensitivity, and scotomas. This will make it easier for schools and families to use correct lighting conditions and contrast. Strong sunlight is often problematic, and sunglasses should be encouraged.

### 7.3. Balance

Bilateral vestibular areflexia in USH1 results in impaired and delayed motor milestones. Thus, early rehabilitation has to focus on making use of somatosensory and visual signals to compensate for vestibular loss. This can be done in preschools and schools by having extra physical training under the guidance of a physiotherapist.

Rehabilitation will benefit from close cooperation between ENT/audiology and ophthalmology/low-vision clinics. Adjustments, especially with special light conditions, must be executed in the home, schools, and later in life, at workplaces.

### 7.4. Physical and Psychological Comorbidity

Recent research in Sweden has shown that adults with USH generally have poor physical health compared with a cross section of the Swedish population. The most prominent physical symptoms were fatigue, headache, as well as neck and shoulder pain. Psychological health was also impaired, with the inability to manage problems and lack of social trust. The USH group showed a statistical over-representation of suicidal behaviors [127,128].

Thus, rehabilitation in USH must focus not only on vision and hearing, but also on the psychosocial consequences of deaf-blindness. Rehabilitation must be multidisciplinary and involve family, school, employer, etc. Rehabilitation is lifelong and increases with age.

It must be noted that the epidemiology presented in Table 2 is uncertain because of significant geographical variations. Furthermore, many factors are poorly studied and range from lack of adequate and correct diagnosis to few countries having national registries and presence of geographical isolates, such as Northern Scandinavia and Louisiana (USA).

## 8. Treatment and Future Perspectives

Gene therapy in USH has undergone significant achievements during the last 5 years. Several different approaches are currently used in different stages of animal and human clinical trials. Most research has focused on the retina and preserving vision. The reasons for this are that CI has been shown to both allow hearing in USH1 and restore hearing in USH2 and 3. Moreover, the retina is easier to access than the cochlea. Nevertheless, gene therapy studies are ongoing and directed towards the retina, cochlea, and vestibular apparatus.

Mice and zebrafish are often used as in vivo animal models. Translating results from animal models is often difficult, but the results from current clinical trials will hopefully result in new treatments. Different approaches have been used, such as adeno-associated viral vectors, for in vivo gene transfer. It has the advantage of using a DNA virus that is nonpathogenic to humans. It appears to enable long-term transduction after a single vector administration and has a good safety profile in humans. Some of the USH genes are quite large, and a potential disadvantage can be limited cargo capacity. Clinical trials using dual AAV vectors have been conducted. Another approach is to use antisense oligonucleotides, with clinical application becoming a reality and several drugs already in trials. Nonsense mutations are common in USH, and the drug Ataluren creates a premature stop signal in the translation of the genetic code, which allows the cell to produce a full-length, functional protein.

The discovery of CRISPR–CAS9 that received the Nobel Prize in 2020 is another method enabling in vitro correction of the c2299delG mutation in the *USH2A* gene. An excellent review of the gene-, drug-, and cell-based therapies has recently been published by French et al. [129].

## 9. Conclusions

USH is one of the most common autosomal recessive syndromes associated with double-sense impairment, hearing, and vision. This combination creates a unique entity of deaf-blindness. Genetics has enabled significant advances, accurate diagnosis, and improved understanding of the pathology and is expected to provide innovative treatments for gene therapy. Next-generation sequencing has also helped in new conceptions and knowledge of the field.

The rehabilitation of these patients is complicated and requires specific knowledge and education. Even if bioengineering provides continuous progression in retinal support, the best treatment option remains hearing rehabilitation with hearing aids and CIs. Bilateral devices can partially restore spatial orientation and support residual visual function. Encouraging results have been published on gene therapy in experimental animal models, but this approach has not been translated into current clinical management. Nevertheless, further studies are necessary to develop new strategies and collect information on USH.

## Figures and Tables

**Table 1:** Genes potentially involved in Usher syndrome (in alphabetic order): nine confirmed causative genes are reported in grey. Names of proteins and genes have been reported according to Uniprot (www.uniprot.org, accessed on 21 December 2021) and GeneCards (www.genecards.org, accessed on 21 December 2021).

Gene	Chr. (Loci)	Protein	Role	Expression	Effect When Mutated	Usher Type	Grade of Evidence
*ABHD12*	*20p11.21*	Lysophosphatidylserine lipase ABHD12 (abhydrolase domain containing 12)	Functional protein (enzyme involved: endocannabinoid system and neurotransmission)	Nervous system, thyroid, retina, skin, nasal epithelium	Neurodegeneration	USH3?	To be confirmed
*ADGRV1*	*5q14.3*	Adhesion G protein-coupled receptor V1	Functional protein G-protein transmembrane receptor. Usher protein complex 2.	Nervous system, eye, and ear.	Ciliary dysfunction. Vision impairment. Familial febrile seizures	USH2C	Confirmed and widely reported
*ARSG*	*17q24.2*	Arylsulfatase G	Functional protein, Enzyme (sulfatase)	Lysosomes	Dysregulation of hormone biosynthesis	USH4?	To be confirmed
*CDH23*	*10q22.1*	Cadherin-23	Structural protein (adhesion protein)	Retina and cochlea; Usher protein complex 1.	Poor intercellular adhesion	USH1D and DFNB12	Confirmed and widely reported
*CEP250*	*20q11.22*	Centrosome-associated protein CEP250	Protein required for centriole-centriole cohesion during interphase of the cell cycle	Ubiquitous, centrosome and cilia body	Centrosomal dysfunctional activity and ciliopathies	atypical USH?	To be confirmed
*CEP78*	*9q21.2*	Centrosomal protein of 78 kDa	Protein required for centriole-centriole cohesion during interphase of the cell cycle	Ubiquitous, centrosome and cilia body	Centrosomal dysfunctional activity and ciliopathies	atypical USH?	To be confirmed
*CIB2*	*15q25.1*	Calcium and integrin-binding family member 2	Intracellular calcium homeostasis	Ubiquitous	Dysfunction in cellular activities	USH1J?	No longer considered an Usher gene
*CLRN1*	*3q25.1*	Clarin-1	Transmembrane protein	Synapses, inner ear, and retina	Interruption of the signal transmission	USH3A	Confirmed
*WHRN*	*9q32*	Whirlin	Gene product is a PDZ scaffold protein expressed in hair cells and photoreceptors	Usher protein complex 2	Profound prelingual deafness; rare cause of recessive deafness and RP	USH2D and DFNB31	Confirmed
*ESPN*	*1p36.31*	Espin	The gene product is an actin-bundling protein that plays a role in transduction in mechanosensory and chemosensory cells	Tip complex	Possible USH with or without vestibular symptoms	USH1M? and DFNB36	To be confirmed
*HARS* *1*	*5q31.3*	Histidine--tRNA ligase, cytoplasmic	Protein-coding gene	Cytoplasmic enzyme that belongs to the class II family of aminoacyl-tRNA synthetases	Possible USH as recessive pattern and Charcot–Marie– Tooth disease as dominant pattern	USH3B?	Its role must be confirmed
*MYO7A*	*11q13.5*	Unconventional myosin-VIIa	Myosin, a structural component of cilia and microvilli, found in several tissues, including inner ear hair cells, photoreceptors, and RPE	Ubiquitous; Usher protein complex 1.	USH and DFNB2	USH1B	Confirmed
*PCDH15*	*10q21.1*	Protocadherin-15	Structural protein involved in tip links (USH1 complex).	Stereocilia in inner ear hair cells and photoreceptors. Usher protein complex 1.	USH and DFNB23	USH1F	Confirmed
*PDZD7*	*10q24.31*	PDZ domain-containing protein 7	It is considered a modifier gene for Usher syndrome.	Usher protein complex 2	It is responsible for autosomal recessive hearing loss DFNB57	USH2?	To be confirmed
*USH1C*	*11p15.1*	Harmonin	Structural protein	Usher protein complex 1	USH and DFNB18	USH1C	Confirmed
*USH1G* *(SANS)*	*17q25.1*	Usher syndrome type-1G protein	Structural scaffold protein	Usher protein complex 1	Usher	USH1G	Confirmed
*USH2A*	*1q41*	Usherin	Structural protein	Usher protein complex 2	Usher	USH2A	Confirmed
?	USH1E *	21q21				?	mapping linkage
?	USH1H *	15q22-q23				?	mapping linkage
?	USH1K *	10p11.21-q21.1				?	mapping linkage

* Unidentified genes; RPE = retinal pigment epithelium.

**Table 2:** Types and subtypes of Usher syndrome and distribution in the general population as estimated * by epidemiological studies. Nine confirmed causative genes are reported in grey. The gene *CIB2* has recently been excluded by the extended research of Booth et al. in 2018. There is some evidence that suggest a role of gene *HARS* in Usher 3B; however, its role must be confirmed in further studies.

Type	Gene	Chr. (Locus)	Protein	Epidemiology * (% Mutations ^a^)	Year of Identification
Usher type I (35–40%)					
IB	*MYO7A*	11q13.5	Myosin Vlla	50–70%	1995
IC	*USH1C*	11p14.3	Harmonin	6–20%	2000
ID	*CDH23*	10q22.1	Cadherin 23	10–20%	2001
IE	Unknown	21q21.3	Unknown	Unknown	*1997*
IF	*PCDH15*	10q21.1	Protocadherin 15	5–10%	2001
IG	*USH1G (SANS)*	17q25.1	Usher syndrome type 1G protein	0–5%	2003
IH	Unknown	15q22-q23	Unknown		2009
IJ	*CIB2 (?)*	15q25.1	CIB2	No longer USH gene	2012
IK	Unknown	10p11.21-q21.1	Unknown	Unknown	?
Usher type II (60–65%)					
IIA	*USH2A*	1q41	Usherin	50–80%	1998
IIC	*ADGRV1 (GPR98)*	*5q14.3*		5–20%	2004
IID	*WHRN (DFNB31)*	9q32	Whirlin	0–10%	2007
Usher type III (0–5%)					
IIIA	*CLRN1*	3q25.1	Clarin-1 transcript variant	90–95%	2001
IIIB	*HARS*	5q31.3	Cytoplasmic histidine--tRNA ligase	5–10%, however its role must be confirmed	?

^a^ Approximated among patients with Usher type I.

**Table 3:** Usher clinical types and main features.

Type	Hearing Loss	Vestibular Function	Retinitis Pigmentosa (RP)	Hearing Rehabilitation and Communication
USH1	Congenital, severe-to-profound sensorineural hearing loss; however, exceptions with moderate hearing loss are reported.	Absent or abnormal vestibular function; however, exceptions are widely reported, from moderate to normal function	Early onset	Bilateral. Good outcomes with early cochlear implantations. Some exceptions have been reported for patients with *PCDH15* mutations. However, further studies are required to confirm this statement. Tactile signing and sign language may be necessary in selected cases.
USH2	Congenital/prelingual sloping down, moderate-to-severe sensorineural hearing loss.	Normal	Late onset, but usually in young/adult age.	Outcomes can be related to the hearing loss onset. Patients with stable hearing loss may benefit from hearing aids. Cochlear implantations have also shown good outcomes. Bilateral. Bimodal rehabilitation should be considered. They may benefit from sign language.
USH3	Variable onset of moderate-to-severe hearing loss potentially fast progressive.	Variable	Variable onset, but essentially during early adulthood	Hearing aids till surgery if indicated. Progression of hearing loss should carefully be monitored. Bilateral rehabilitation. Due to progression, if indicated, bilateral cochlear implantation is a valid option, but bimodal stimulation can also be effective. Efficacy of sign language in selected cases depends on onset and progression.

## Data Availability

Not applicable.
